# Study of mandible reconstruction using a fibula flap with application of additive manufacturing technology

**DOI:** 10.1186/1475-925X-13-57

**Published:** 2014-05-06

**Authors:** Ming-June Tsai, Ching-Tsai Wu

**Affiliations:** 1 Mechanical Engineering Department, Cheng Kung University, No.1, University Road, Tainan 701, Taiwan

**Keywords:** Additive manufacturing, Computer-aided engineering, Mandible reconstruction, Rapid manufacturing, Reverse engineering

## Abstract

**Background:**

This study aimed to establish surgical guiding techniques for completing mandible lesion resection and reconstruction of the mandible defect area with fibula sections in one surgery by applying additive manufacturing technology, which can reduce the surgical duration and enhance the surgical accuracy and success rate.

**Methods:**

A computer assisted mandible reconstruction planning (CAMRP) program was used to calculate the optimal cutting length and number of fibula pieces and design the fixtures for mandible cutting, registration, and arrangement of the fibula segments. The mandible cutting and registering fixtures were then generated using an additive manufacturing system. The CAMRP calculated the optimal fibula cutting length and number of segments based on the location and length of the defective portion of the mandible. The mandible cutting jig was generated according to the boundary surface of the lesion resection on the mandible STL model. The fibular cutting fixture was based on the length of each segment, and the registered fixture was used to quickly arrange the fibula pieces into the shape of the defect area. In this study, the mandibular lesion was reconstructed using registered fibular sections in one step, and the method is very easy to perform.

**Results and conclusion:**

The application of additive manufacturing technology provided customized models and the cutting fixtures and registered fixtures, which can improve the efficiency of clinical application. This study showed that the cutting fixture helped to rapidly complete lesion resection and fibula cutting, and the registered fixture enabled arrangement of the fibula pieces and allowed completion of the mandible reconstruction in a timely manner. Our method can overcome the disadvantages of traditional surgery, which requires a long and different course of treatment and is liable to cause error. With the help of optimal cutting planning by the CAMRP and the 3D printed mandible resection jig and fibula cutting fixture, this all-in-one process of mandible reconstruction furnishes many benefits in this field by enhancing the accuracy of surgery, shortening the operation duration, reducing the surgical risk, and resulting in a better mandible appearance of the patients after surgery.

## Background

Lesion resection is often used to remove mandible lesions in the clinical setting, but results in a large-area mandible defect. A surgical approach is used to reconstruct mandibular bony defects. There are at least three different surgical approaches that can be used for reconstructing mandible defects, including free fibula flap, local flap, and bone plates with screws. During reconstruction by free fibula flap, skin and a portion of fibula in the adjoining soft tissue are harvested when the fibula is removed, and the fibula and soft tissues are used to restore the defect by microscopic anastomosis surgery to allow integration with the arteries and veins of the neck [1]. At present, this method is the most prevalent for mandible reconstruction surgery, because the length of the fibula is adequate for the reconstruction of various mandible defects, and in addition the survival rate is up to 90% [2-4]. Traditionally, surgeons harvest an amount of bone and a proper amount of adjoining soft tissues sufficient to restore the defect area. The fibula is then cut into several pieces, and the small pieces of fibula are arranged to restore the mandibular bony defect and fixed by plates and screws. However, the shape of mandible is resemblance to a circular arc, which is quite different from the geometrically linear shape of the fibula. How to cut the fibular bone into pieces to pile up the original mandible shape is a big problem for doctors. Therefore, it is often the case that reconstruction results in an asymmetric shape of the mandibular bone. This not only causes a cosmetic problem, but also leads to a greater challenge in terms of the design of dental implants and dentures in the future.

Several methods have been proposed in the literature to solve the above-mentioned problems. Due to recent progression in technology, computer assisted surgical planning has become prevalent. Heather et al. [5] applied Virtual surgical planning and Surgical design and simulation to real five cases study with results of obvious benefits. The application of imaging analysis of the data on the three-dimensional orientation of the bone segments during the preoperative planning, which can increase the treatment efficiency [6]. Hallerman et al. [7] and Yeung et al. [8] demonstrated that reverse engineering can be used to construct a mandible digital model and rapid prototyping can promptly generate a mandible model as the preoperative reference. The preplating technique proposed by Marchetti et al. [9] and Ciocca et al. [10] are useful methods for oromandibular reconstruction that can result in good occlusal and functional rehabilitation. Other studies have also reported the preparation of a customized titanium plate for the surgical area before tumor resection, and once the tumor has been removed, the plate can be used as a template for arranging the segments of fibula [11-13]. Rohner et al. [14] and Kernan et al. [15] suggested designing a cutting template based on the occlusal position, which allows the doctor to cut the fibula along the template. However, as the cutting template needs to be prepared before the operation, it is difficult to modify if any changes are required during the operation.

Among the above-described methods, application of the medical imaging method can reconstruct a 3D digital model and provide a reference model of the mandible before surgery. However, it does not generate a template as a reference for the fibula, and clinicians need to measure manually the amount of bone required and then consider how to cut and arrange the fibula segments. This often prolongs the process of preoperative preparation and might cause errors due to the lower accuracy of manual measurement. A premade cutting fixture is often not able to fit the defect area due to lesion resection being difficult to predict. Many fixtures cannot be manufactured and applied in a timely fashion during surgery. Application of a metal bone plate provides a good reconstruction target for the mandible defect, but it cannot function effectively as a template for fibula cutting and as a positioning tool for reconstruction.

To overcome the above-mentioned deficiencies, this study first established digital models of the mandible and fibula with the corresponding nodes (locations) for cutting planes (CPs) according to the medical images. The simulation design for the lesion resection was created on the basis of the sequential CP point group. The CAMRP [16,17] generated by our research group was used to calculate the optimal lengths and number of fibula pieces, based on a digital mandible model for defect resection and reconstruction using the fibula bone. Design of the cutting jig and the registering fixture were rapidly generated according to the preoperative digital model, and the designed cutting jig has many guide slots which could be chosen if the resection conditions changed during the procedure. The digital models for all the design models, including cutting jigs and registered fixtures, can then be simultaneously made using additive manufacturing (AM) technology prior to the operation. This provided all the necessary requirements for every step of the mandible defect reconstruction process. This study established a technique for lesion resection that can simultaneously complete fibula cutting and mandible defect reconstruction surgery in one operation. The technique can significantly shorten the duration of surgery, reduce the number of the visits for patients, and enhance the accuracy and success rate of the surgery.

## Methods

**Figure 1:**
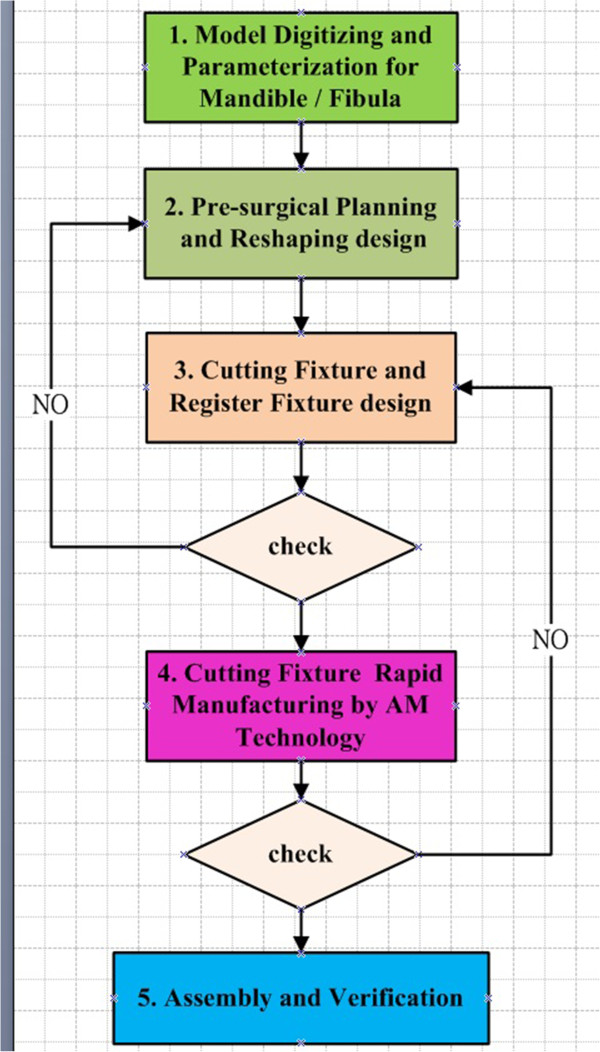
Study flowchart.

The flow chart of the study was as shown in Figure 1, which includes five main processes: model digitizing and parameterization, pre-surgical planning and design, cutting fixture design, cutting fixture rapid manufacturing, and assembly and verification. Below is an explanation of each process.

### Digitized and parameterized models of the mandible and fibula

**Figure 2:**
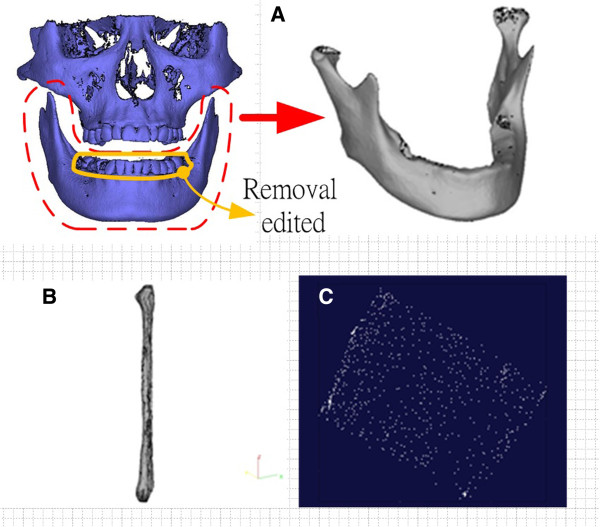
**Digital model of the mandible and fibula. (A)** STL model of the skull and the mandible, **(B)** STL model of the fibula, **(C)** random point cloud of the scanned image.

In this study, the digital model of lesion mandible was designed and captured from a skull model which was obtained by 3D tomography from a healthy man. The 3D tomography images which came from an adults sponsor in our previous study approved by IRB (EMRP01099N) of E-DA hospital Taiwan. The data of skull were then manipulated using MIMICS software (version 13.0) to generate a stereolithography (STL) digital file (Figure 2A, left). In order to generate a mandible lesion area, 3Matics software was used to design the lesion area by removing the teeth on the model, which created a mandible digital model (Figure 2A, right). In addition, a digital model of the fibula (Figure 2B) was captured from an artificial teaching sample using a high-resolution scanner (Germany, ATOS 3D SCANNER).

**Figure 3:**
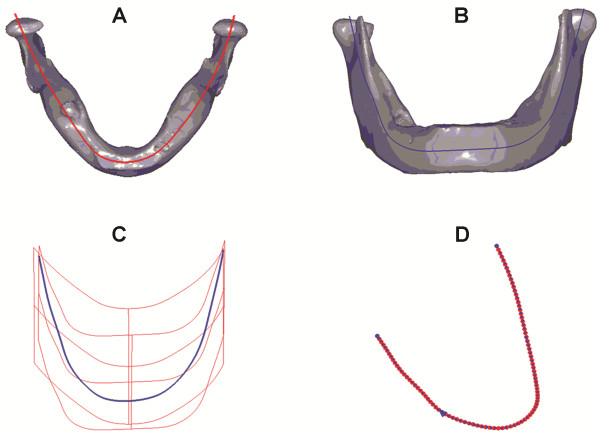
**Center line design of the mandible and fibula. (A)** The center line of the top view; **(B)** The center line of the front view; **(C)** Generation of the reference center line for the mandible; **(D)** The 101 node of the center line.

As the data retrieved from imaging scanning were of a random point cloud, as shown in Figure 2C, we used a process to parameterize non-ordered data in the scanned image. The process used Surfacer (version10.0), a reverse engineering software, to calculate the middle lines of the projected outline from the top view and front view of the mandible (Figure 3A and B). The middle line planes were obtained from the middle lines of the top view and front view of the mandible, and an intersecting line of the two middle line planes was generated (Figure 3C). The intersecting line is defined as the reference central line of the mandible, which is called the “center line” for short.

**Figure 4:**
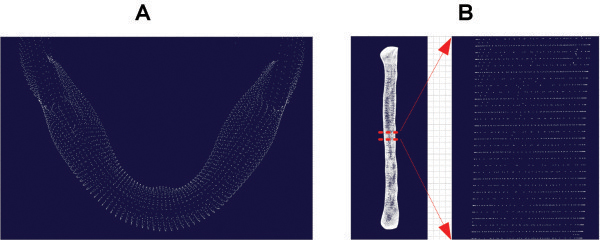
Parameterized model: (A) Parameterized mandible model; (B) Parameterized fibula model.

The center line was partitioned into 100 isometric segments. The point coordinates of all the segments were calculated and 101 node coordinates were obtained in total (Figure 3D). According to the 101 nodes of the mandible center line, the normal vector plane going through all the nodes, which was defined as the CP, was calculated. Then, the intersection of the CP and the mandible STL model outline was calculated to gain the points cloud of 101 CPs of the mandible, and according to the points cloud group of these 101 CPs, a parameterized mandible digital model (Figure 4A) was reconstructed. Thus, this parameterized model was combined from the point cloud of the 101 CPs. According to the CPs going through every node, cutting calculation of the mandible can be carried out. The same method was used to construct the parameterized digital model of the fibula. Figure 4B shows the parameterized digital model of the fibula with clear node CPs. Creating a model with sequenced nodes could improve the efficiency of the cutting simulation.

### Pre-surgical planning and shape design

**Figure 5:**
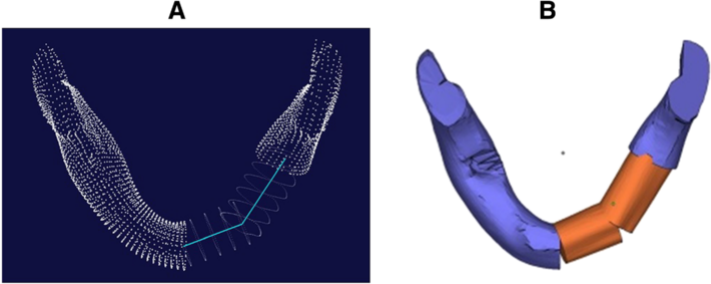
Calculation by an optimal program and the design of model for mandible defect area reconstruction: (A) The case of two pieces of fibula; (B) The cut mandible model with two pieces of fibula combined.

By using the parameterized mandible model, the lesion area to be removed was planned based on the planes corresponding to each numbered node. An optimal program was used to calculate the cutting length, the number of the fibula pieces required and the best arrangement under different lesion sizes. Figure 5A shows the results calculated by the optimal program, in which 3 pieces of fibula were arranged in the defect area under different defect conditions. Figure 5B shows the designs of the mandible reconstruction digital models that contain 3 pieces of fibula corresponding to the different defect conditions shown in Figure 5A.

### Design of the cutting fixture and registered fixture

Using the design of the optimal program shown in Figure 5, the cutting fixture for the mandible lesion and the registered fixture for the arrangement of fibula pieces were created to improve the surgical accuracy and efficiency. As a perfect match between the mandible cutting fixture and the surface of the mandible is critical, this study retrieved the points cloud data of the mandible defect area and utilized the data to reconstruct the pad of the cutting fixture. Therefore, the fixture can completely fit onto the surface of the mandible. In order to deal with possible variation of the lesion area found during surgery, the mandible cutting fixture was designed with three cutting grooves, which allowed the cutting to be more flexible when a larger or smaller lesion area was found during the operation.

**Figure 6:**
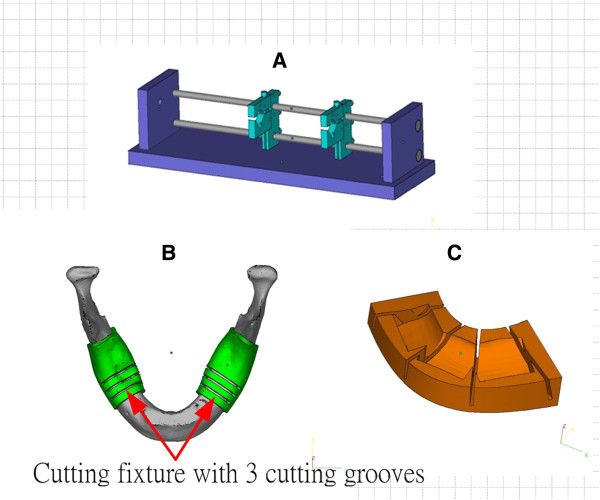
**Design of the cutting fixtures and registered fixtures. (A)** Fibula cutting fixture; **(B)** Cutting fixtures for area mandible lesions; **(C)** Registered fixtures for the arrangement of cut fibula pieces.

The design of the cutting fixture was made according to the required cutting length and the number of pieces of fibula, and the cutting face was vertical to the center line of the fibula. The design can make the cutting simple to perform, so fibula cutting can be completed in a short amount of time. Figure 6 shows the design of the cutting fixtures of the fibula and mandible, in which Figure 6A is the fibula cutting fixture that presets the cutting length according to the different cutting lengths and positions of the pieces of fibula. Figure 6B show the 3D designs of the mandible cutting fixtures. As the internal surface of the cutting fixture perfectly matches the surface of the mandible, the cutting fixture can quickly be fitted onto the position of the mandible lesion to be removed. Figure 6B show the designs of fixtures with 2–3 cutter grooves, which could be adapted to the needs of flexible resection if the area of the mandible defect is larger or smaller than postoperatively predicted.

After completion of the cutting of the mandible lesion model and fibula, the pieces of the fibula need to be arranged and positioned into the space between the two cutting ends of the mandible defect. In this study, a registered fixture was designed using Boolean algebra to calculate the optimal combination of fibula pieces. The registered fixture can help to rapidly arrange the fibula pieces onto the defect area (Figure 5), satisfying the clinical needs for mandible defect reconstruction. Figure 6C shows the registered fixtures for fibula arrangements adapted for 3 pieces of lesion conditions.

### Application of Additive Manufacturing (AM) for cutting of the entity

The completed mandible model, fibula model, cutting fixture and registered fixture are digital models with complicated geometric characteristics. AM technology was applied to rapidly output the entities of the cutting fixture and registered fixture to meet the customized requirements for clinical application in a timely manner. The mandible cutting fixture can be used to fit onto the mandible for rapid and accurate cutting in the clinic, and the registered fixture can ensure that the cut fibula pieces are rapidly positioned and arranged, which can allow mandible reconstruction surgery to be completed in a short time.

### Verification and assembly

The error values of the design files for the cutting fixture and registered fixture were verified using reverse engineering software. To ensure the accuracy of the model, the error of the digital model was controlled to within 0.1 mm. and the physical model of fixture was controlled to within 0.5 mm. The fibula entity, after being output by the AM system, was cut using the fibula cutting fixture. The cut fibula pieces were installed onto the registered fixture and then placed onto the defect area of the mandible. The results showed that this technique can achieve the target of mandible reconstruction.

### All-in-one mandible reconstruction process

**Figure 7:**
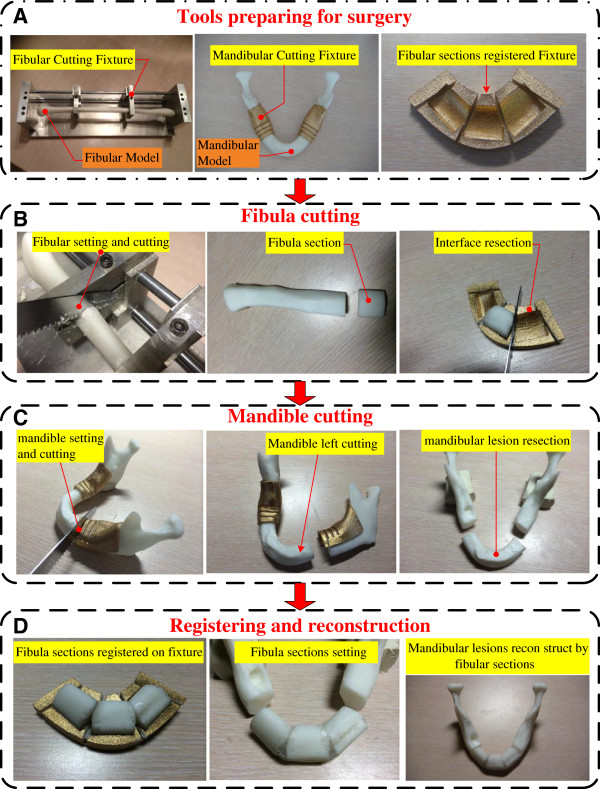
One-step mandible reconstruction process: (A) tools in preparation for surgery; (B) fibula cutting process; (C) mandible cutting process; (D) registering and reconstruction.

After completing the design, cutting planning and registration process, the cutting jig and registered fixture were prepared by AM systems before surgery. Figure 7 shows the detailed process of cutting, registering, and mandibular reconstruction. First, the defected mandible STL model was input into the CAMRP, and the doctor decided on the location for lesion resection. Then, the CAMRP calculated the optimal length and pieces of fibula for cutting. The custom-made mandible cutting guide-jigs were generated from the neighboring surfaces of the cutting locations. Meanwhile, the number and length of fibular segments were known, and the registering fixture was ready for surgery (Figure 7A). The second step was fibula cutting according to the physical guiding fixtures. The harvested fibula bone was set and cut on the alumina alloy fixture based on the length and number of sections according to the planning of the CAMRP (Figure 7B). Third, mandibular cutting guide-jigs were snap-fitted, neighboring the lesion area, for resection according to the surgical planning (Figure 7C). Finally, the doctor placed the fibula pieces on the registering fixture at the designed position to shape the mandible (Figure 7D). If the initial resection was not long enough, the guide-jigs provided some more slots for cutting, and the new cutting location was input into the CAMRP for re-planning of the cutting of the fibular bone. The process of this study, which removes and reconstructs the mandibular lesion using fibular bone segments in one step, is easy to reproduce in clinical practice.

## Results

**Figure 8:**
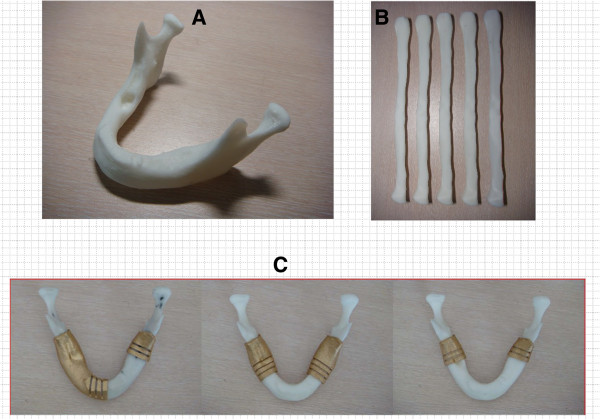
The entity models made by AM and the cutting fixtures: (A) the mandible entity model; (B) the fibula entity model; (C) the mandible lesion cutting fixture.

Reverse engineering technology and computer tomography were applied to reconstruct the digital model in this study (as described in Figure 2). Figure 6 shows the design of the cutting fixture and the registered fixture. Figure 8 shows the detail of the mandible and fibula model entities outputs by the AM system as well the cutting fixture and registered fixture. The mandible and fibula entity models are useful objects for planning, design, discussion and measurement (Figure 8A,B). The fibula cutting fixture is shown in Figure 7, and Figure 8C shows the AM parts of mandible lesion cutting fixture with 2–3 cutter grooves set up on the mandible model, which is used during lesion removal.

**Figure 9:**
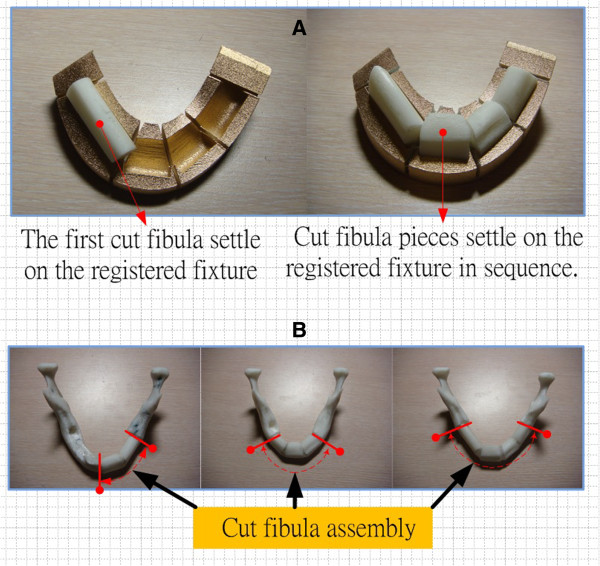
**Integrated positioning of the fibula pieces and the completed mandible defect reconstruction model. (A)** to position the fibula pieces on the registered fixture; **(B)** the assembly of fibula pieces for the completed mandibular reconstruction.

The fibula was placed on the fibula cutting fixture to cut it into suitable pieces, and the cut fibula pieces were then arranged on the registered fixture in sequence (see Figure 9A). After the position orientation was completed based on registered fixture, the fibula assembly was fixed and available to be quickly set up onto the mandible defect area for completion of the mandible reconstruction entity model. Figure 9B shows the AM parts of the mandible reconstruction physical objects that contain 2-4 pieces of AM parts of fibula corresponding to the different defect conditions.

**Figure 10:**
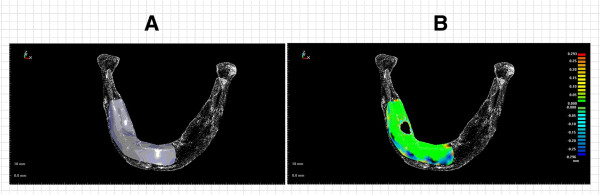
**The digital model of mandible cutting fixture and error analysis. (A)** The cutting benchmark curved surface of the right side mandible lesion; **(B)** Comparison of the range error of the right side cutting fixture.

**Figure 11:**
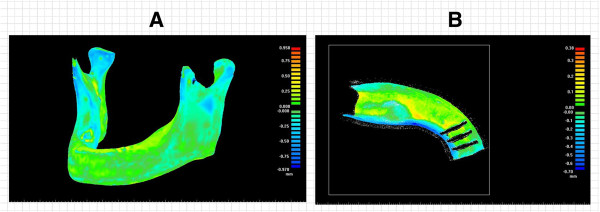
**The real physical model of mandible cutting fixture and error analysis. (A)** The error map of the physical model of mandible; **(B)** The error map of the physical model of mandible cutting fixture.

The error analysis of designed digital model was shown as Figure 10. Figure 10A show the right curved surfaces of the cutting fixture for resection of the mandible lesion area, which is the foundation of the mandible cutting fixture. The design of the benchmark curved surface of the cutting fixture was compared with the tomography point data of the original mandible to clarify the range error. Figure 10B are graphs of the range differences between the designed benchmark curved surface of the cutting fixture and the tomography point data of the original mandible. The maximum and average errors of digital model are as shown in Table 1, which indicates that the maximum error of digital design model was 0.30 mm and the average error of digital model was 0.03 mm. The error map analysis of real model was shown as Figure 11. Figure 11A show the graphs of the range differences between the digital model of mandible and the scanning point data of the real model of mandible. Figure 11B are error map of the range differences between the digital model of cutting fixture and the scanning point data of the real model of cutting fixture. The maximum error and average of real model of mandible and fixture are as shown in Table 2. The maximum error of real model of mandible was 0.97 mm and average error of real model of mandible was 0.13 mm. The maximum error of real model of cutting fixture was 0.70 mm and average error of real model of mandible was 0.15 mm.

**Table 1:** Comparison of the errors of the digital model of the mandible cutting fixture

**Item**	**Error (Max.)**	**Error (Avg.)**	**Standard deviation**
Right side cutting fixture curved surface	0.30 mm	0.03 mm	0.06 mm

**Table 2:** Comparison of the errors of the real model of mandible and cutting fixture

**Item**	**Error (Max.)**	**Error (Avg.)**	**Standard deviation**
The real model of mandible	0.97 mm	0.130 mm	0.11 mm
The real model of cutting fixture	0.70 mm	0.15 mm	0.16 mm

## Discussion

In traditional mandible reconstruction surgery, several segments of the fibula are harvested from the patient and are normally manually arranged onto the defect area after the mandible lesion is removed. However, due to the shape of the fibula being linear and quite different from the curved shape of the mandible, the reconstructed mandible appearance of the patient is often asymmetric or causes cosmetic issues. In addition, the large difference in the geometric shapes can cause difficulties when dental implants and dentures are installed. In this study, we used advance medical imaging and reverse engineering technology to obtain digital models for patients as the basis of a parameterized digital model, and applied the technique to speed up the design prior to the surgical procedure.

The six steps to fulfill the mandibular reconstruction postulated by this study, which can easily be reproduced and is suitable for clinical practice or other application, are as follows:

1. Input the patient’s 3D mandible model into the CAMRP. The doctor then decides the cutting nodes to remove the lesion portion.

2. The CAMRP performs surgical planning and generates 3D models of the cutting jigs and fixture.

3. Output the physical cutting jigs and fixture by 3D printing technology.

4. Use the guiding jigs to cut the ridus portion of the mandible.

5. Harvest the fibula, place it on the fixture, and cut into pieces based on the lengths computed by the CAMRP.

6. Assemble the fibular segments into a registering fixture for mandibular reconstruction.

The periosteum and pedicles are very important in the surgery. In the real cases, we could design a tray under the clamping parts of fibula cutting fixture to support the periosteum and pedicles parts of fibula cutting. The clamping parts will limit the cutting direction, so we need to rotate the fibula bone by centerline of axis to the proper direction where can easy to fulfill the cutting step. This step is very important to avoid of damage of periosteum or pedicles by gravity loading and external force in the surgery process. The design of the fibula cutting fixture should be simplified as much as possible, so as to allow the doctor to rapidly position the fibula and cut it accurately, while the design of the mandible lesion cutting fixture should be as flexible as possible. Therefore, the fixture can be used flexibly, as the lesion might be larger or smaller than predicted, which can only be found when surgery is performed. The digital files of the fibula and mandible cutting fixtures were used to rapidly output the entity fixtures through the AM system. This provided a useful reference for the design of the cutting fixture and registered fixture, which are key in this study. The results of this study demonstrated that our proposed method can help to complete mandible lesion resection and fibula defect reconstruction in one single surgery, and is expected to achieve a good effect in clinical application.

**Figure 12:**
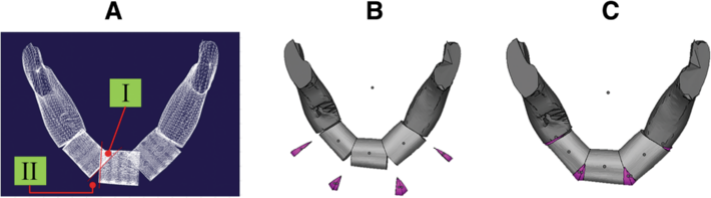
Overlapped areas of fibula pieces during assembly: (A) Interference of the overlapped bone; (B) removed corner bone (purple); (C) final mandible lesion reconstruction filled up with the small pieces.

The CP of the fibula cutting fixture was designed to be vertical to the center axis of the fixture. In order to improve the outcome of the arrangement of fibula pieces, the interference at the corner of the bone needs to be removed (overlapped area of Area I in Figure 12). Thus, our registered fixture has the function to remove the corner of overlapped bone, which makes clinical mandible defect reconstruction surgery more convenient (groove design for cutting in Figure 7). In addition, the removed corner can be used to fill the gap of Area II (Figure 12A), which can make the fibula pieces connect better and lead to the reconstruction being more successful. Figure 12B and C show the process and completion of the reconstruction model by filling up with the small bone pieces removed from Area I.

A universal design of based surface can be designed for the mandible cutting fixture, which would fit very closely with the mandible surface (Figure 10A,B). This surface can be used to design cutting grooves to adapt to the cutting needs of different cases, which can save a lot of time in terms of redesign of the cutting fixtures (Figure 7). The design of the cutting fixtures is based on this universal based surface, and the curved surface is designed to stretch over the two sides of the mandible bone ridge, with the curved surface extension depth of the lingual side being controlled to within 2 mm. The curved surface extension depth of the buccal side should not be over half of the front view height of the mandible, thus avoiding failure of installation of the cutting fixture owing to interference.

The results of the Boolean calculation for the fibula optimal assembly can be applied to design and arrange the registered fixture, and also to install the fibula pieces in the defect area. This can help to rapidly position the fibula pieces in an optimal sequence, and so can increase the surgical accuracy and shorten the surgical duration.

Recently, the medical image modeling and AM medical tooling are well service for the planning, design, simulation and manufacturing of pre-surgery and clinical application [18-20]. Not only the commonplace application of design and simulation of pre-surgery of fibular and mandible cutting surgery, but also we provided the real cutting and setting fixture by AM system which are essential of fulfilling one-stop solution processing of mandible reconstruction of this research. In this study, the fibula cutting fixture was designed from lightweight aluminum, which reduced the weight and increased the convenience for clinical application. Aluminum products could be used repeatedly after autoclave sterilization. The mandible fixtures were made of very strong and elastic nylon material. In terms of practical application, there are more and more material options as the AM system is maturing [21,22]. In recent years, AM technology has been used to output materials that the human body is able to absorb (such as PLA) or materials of human body affinity (such as titanium alloy). Therefore, registered fixtures made of these materials can stay in the human body, and many surgical procedures can be made shorter and simpler.

This study is belonging to simulation of pre-surgery, while not yet enter the process of human subject research. After the processing of digital design, simulation, manufacturing, assembly and testing, the results of this study reveal the high feasibility and effectiveness in the real clinical application. We will plan an advance clinical surgery researching in the near future for the purpose of this study.

## Conclusions

In this study, we created the processes of one-stop solution of mandible construction which the surgical processes for patients with oral cancer were simulated, including completion of mandible lesion resection, preparing fibula pieces, recombining the fibula and completion of the mandible defect reconstruction. The designed models show that this advanced method can achieve a high accuracy with the help of a guiding-jig. This reduces the stress on the doctor during surgery, and the surgical procedure can be completed within a shorter time. The application of computer-aided engineering as well as advanced AM technology allows all these processes to be fulfilled in one operation. The technique established in this study can be applied in clinics to greatly reduce the duration of surgery, shorten the course of treatment, and decrease the number of return visits for patients. It can significantly increase the accuracy and success rate of mandible reconstruction surgery, as well as give patients a better facial appearance, as a symmetrical mandible can be easily achieved.

## Abbreviations

AM: Additive manufacturing; STL: Stereolithography; CP: Cutting plane.

## Competing interests

The authors declare that they have no competing interests.

## Authors’ contributions

MJT initialized the original concept of this study and the optimal program for the mandible reconstruction using a fibula flap. CTW participated in the design of the study and performed the data analysis. Both authors read and approved the final manuscript.
